# A Rare Case of Popliteal Venous Aneurysm

**DOI:** 10.1155/2010/579256

**Published:** 2010-03-10

**Authors:** Roberto Fiori, Roberto Chiappa, Eleonora Gaspari, Giovanni Simonetti

**Affiliations:** Department of Diagnostic Imaging, Molecular Imaging, Interventional Radiology and Radiation Therapy, University Hospital “Tor Vergata”, 81 Oxford Street, 00133 Rome, Italy

## Abstract

We report a case of a 21-year-old man with a popliteal venous aneurysm of the left popliteal fossa, with local symptoms and pain during palpation. Early diagnosis is fundamental in order to prevent the thromboembolic events or other major complications. Duplex scanning, Computed Tomography scanning, and Magnetic Resonance imaging are considered to be important non-invasive diagnostic methods for the diagnosis of PVA. The Angio Computed Tomography acquisition confirmed a 36 mm × 17 mm oval mass in the left popliteal fossa continuous with the popliteal veins. This lesion had presented contrast enhancement only in delayed acquisition (180 sec) and so appeared to be a true venous aneurysm and no arterial. The PVA was repaired surgically via a posterior approach to the popliteal fossa. A 4 × 2 aneurysm was identified. In the same time open tangential aneurysmectomy and lateral vein reconstruction were realised. This case is interesting because the Angio Computed Tomography study, in delayed acquisition, has allowed a correct diagnostic assessment of PVA and the surgical treatment.

## 1. Introduction

Popliteal venous aneurysms (PVAs) may cause fatal complications (e.g., pulmonary embolism and other thromboembolic episodes) [1, 2] if they remain undiagnosed or untreated. These lesions may have more or less symptomatic manifestation above all in females and more frequently in people over 40 years [3–6]. We report a case of a 21-year-old man with a popliteal venous aneurysm of the left popliteal fossa, with local symptoms and pain during palpation. 

## 2. Case Report

A 21-year-old male footballer has presented, for a few months after trauma a local discomfort in particular in the left popliteal fossa during palpation and usual daily activities. 

Physical examination was negative for the presence of a soft mass in the upper part of the popliteal left fossa without local signs of inflammation. Instead was positive for the pain in the front part of the left knee during palpation. There was no evidence of clinical signs of peripheral arterial angiopathy. Chest and abdomen examination, arterial blood pressure, chest X-ray, oxygen saturation, and electrocardiogram were normal. 

Duplex scanning, Computed Tomography scanning, and Magnetic Resonance imaging (MRI) are considered to be important non-invasive diagnostic methods for the diagnosis of PVA. 

**Figure 1:**
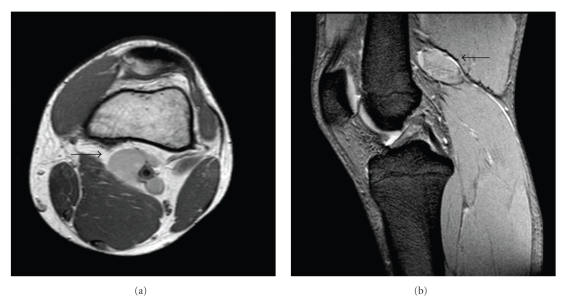
*Magnetic Resonance* (MRI), SE T1 Dual axial (a), SE T2 FFE sagittal (b) showing a 4 cm oval-shaped formation with dishomogeneous hyperintensity in the left popliteal fossa adjacent to the vessels.

Magnetic Resonance imaging (MRI) without contrast media demonstrated a 4 × 2 cm oval formation with dishomogeneous hyperintensity on T1-and T2-weighted sequence in the left popliteal fossa adjacent to the vessels suspected for a blood lesion (Figure 1). 

Then the color-Doppler ultrasonography (US-CD) was performed to identify the true origins of this lesion. This examination demonstrated only a mixed echogenicity mass in the left popliteal fossa with no evidence of arterial or venous nature. 

Subsequently this mass was evaluated with Angio-Computed Tomography (ACT) using a computed tomography scanner (Highspeed Advantage, GE Medical System Milwaukee, WI, USA). 

Images were acquired without and with contrast media intravenously (a single bolus of 120 cc with a flow rate of 3.5 ml/s of nonionic contrast medium via an antecubital venous access) and a scan was obtained with three phases from the injection of contrast media. 

Every data acquired were transferred to an Advantage Windows workstation (GE Advantage) and reconstrued using maximal intensity projection (MIP) and volume rendering (VR) technique. 

**Figure 2:**
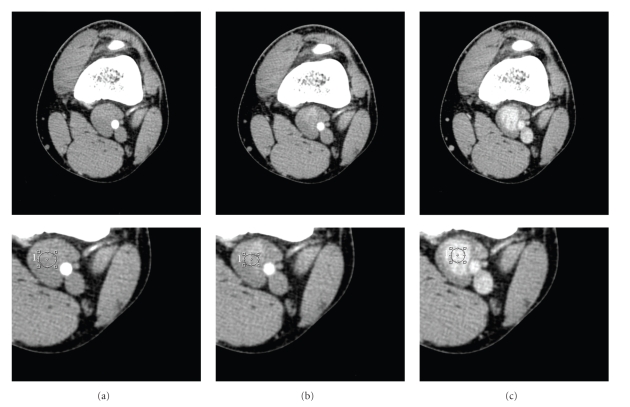
*Angio-Computed Tomography* (ACT) was obtained with three phases: arterious (a), venous (b), and delayed (c) from the injection of contrast media. The ACT acquisition confirmed a 36 mm × 17 mm oval mass in the left popliteal fossa. This lesion presented contrast enhancement only in delayed acquisition (c) and so appeared to be a true venous aneurysm.

**Figure 3:**
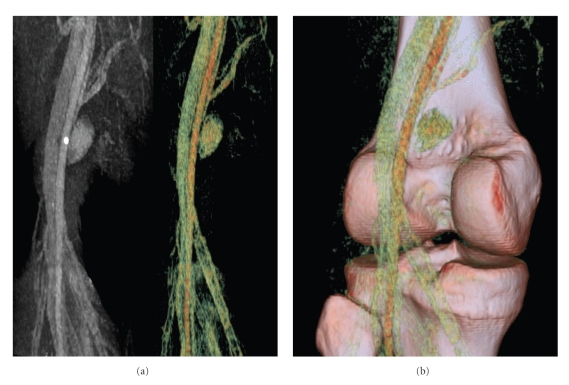
*Angio-Computed Tomography* (ACT) scan reconstruction using maximal intensity projection (MIP), volume rendering (VR) technique, and 3D showing the popliteal venous aneurysm in the left popliteal fossa.

The ACT acquisition confirmed a 36 mm × 17 mm oval mass in the left popliteal fossa continuous with the popliteal veins. This lesion had presented contrast enhancement only in delayed acquisition (180 sec) and so appeared to be a true venous aneurysm(Figures 2 and 3).

**Figure 4:**
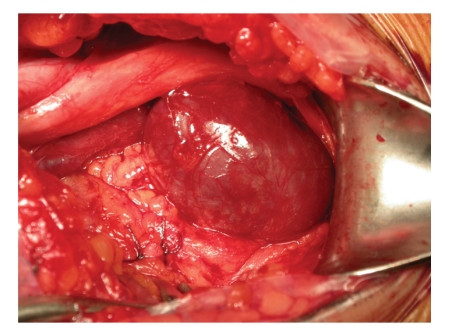
Surgical image showing the popliteal venous aneurysm.

The PVA was repaired surgically via a posterior approach to the popliteal fossa. A 4 × 2 cm aneurysm was identified. In the same time open tangential aneurysmectomy and lateral vein reconstruction were realised (Figure 4). 

A histopathologic examination revealed an aneurysm venous wall with none evidence of inflammatory or degenerative signs. 

The patient was given warfarin for anticoagulation and was discharged on the 3th postoperative day. Two months postoperatively, the patient's symptoms were improved; the repetition of venous duplex scanning showed patency of the repaired popliteal vein without venous thrombosis. 

Elastic stockings were suggested for support, and daily aspirin was recommended for prophylaxis against thrombosis. The patient was able to resume normal activities.

## 3. Discussion

Popliteal venous aneurysms are uncommon and not clinically important in most cases. However they can lead to severe complications including deep-vein thrombosis, pulmonary embolism, and death [7, 8]. 

The etiology of venous aneurismal remains unknown but congenital factors, inflammation, trauma, or degenerative changes have been proposed [9]. Probably in this case repeated episodes of microtrauma related to the footballer contributed to the development of the venous aneurysm. 

Asymptomatic incidental detection, local lower extremity symptoms, or embolic pulmonary episodes may represent different aspects of manifestation of the same condition [1]. 

Most popliteal venous aneurysms are showed with pulmonary embolism (24%) or chronic venous disease (76% limb swelling, postthrombotic symptoms) [10]; but usually they are impalpable on clinical examination, although presentation with a popliteal mass is not uncommon (26%) [11]. 

In this case the ACT study has permitted a correct diagnosis assessment of PVA. This lesion had presented contrast-enhancement only in delayed acquisition (180 seconds) and it had demonstrated that this aneurysm was venous and not arterial. 

Other differential diagnosis of PVA could be the Baker's cyst and tibiofibular cysts. These lesions are different in site and morphology, but the diagnosis is easy with the Ultrasonography and MRI. 

Baker's cyst is a persistent joint fluid effusion (synovial) that can be localized in the back of the knee. It can be caused, more frequently in adults, by posterior herniation of the knee joint capsule. Cysts in the proximal tibiofibular joint are uncommon, and they have a similar manifestation. Their clinical diagnosis is difficult. In both cases the US examination shows an ipo-echogenicity mass and the MRI demonstrates an oval mass hypointensity on T1-weighted sequence and hyperintensity on T2-weighted sequence. 

Surgical options in the treatment of symptomatic and asymptomatic cases include tangential aneurysmectomy with lateral venorrhaphy, resection with end-to-end anastomosis, resection with interposition graft, or ligation of the proximal and distal vein. Early patency rates are encouraging, with no reports of recurrent pulmonary embolism following surgical repair, although the long-term results of surgery are unknown [12].

## 4. Conclusion

Early diagnosis is fundamental in order to prevent the thromboembolic events or other major complications. This case is interesting because the ACT study, in delayed acquisition, has allowed a correct diagnostic assessment of PVA and the surgical treatment.
